# Identification of hsa_circ_0039053 as an up-regulated and oncogenic circRNA in hepatocellular carcinoma via the miR-637-mediated USP21 activation

**DOI:** 10.7150/jca.48998

**Published:** 2020-10-08

**Authors:** Tian Bao Yang, Fang Yi, Wei Feng Liu, Yan Hui Yang, Cheng Yang, Junjun Sun

**Affiliations:** Department of Hepatobiliary Surgery, The First Affiliated Hospital, and College of Clinical Medicine of Henan University of Science and Technology, Luoyang, China, 471003.

**Keywords:** hepatocellular carcinoma, hsa_circ_0039053, miR-637, USP21, ceRNA

## Abstract

Accumulating evidence indicated that circular RNAs (circRNAs) are crucial regulators in tumorigenesis of hepatocellular carcinoma (HCC), but it is still unclear how hsa_circ_0039053 causes HCC. Herein, hsa_circ_0039053 was upregulated in HCC tissues and cell lines. The upregulation of hsa_circ_0039053 was linked to the advanced clinical characteristics of patients. Downregulation of hsa_circ_0039053 decreased the invasion and proliferative ability of tumors *in vitro* and as well as tumor growth *in vivo*. Mechanically, hsa_circ_0039053 positively regulated USP21 expression through interacting with miR-637. Moreover, overexpression of USP21 or silencing of miR-637 restored the inhibitory impacts of hsa_circ_0039053 silencing on HCC progression. Collectively, our study confirmed that hsa_circ_0039053 could be regarded as a competing endogenous RNA (ceRNA) to positively modulate the expression of USP21 combining with miR-637, which provided a potential target in HCC treatment.

## Introduction

Hepatocellular carcinoma (HCC) is estimated to be among the major cancers contributing to tumor-associated deaths worldwide 1, 2. Some main risk factors, including alcohol consumption, obesity, chronic hepatitis B, and C infection, were identified to contribute to the complex process of liver tumorigenesis 3. Although diagnosis and treatments are continuously improved and optimized, the overall five-year survival rate for HCC patients remains low (less than 20%) 4, 5. Therefore, there is a need to explore the molecular mechanism behind HCC tumorigenesis to improve the treatment of the disease.

Circular RNAs (circRNAs) are a class of newly discovered non-coding RNA (ncRNA), characterized by a 3' polyadenylated tail and a covalently closed loop lacking a 5' cap 6, 7. Mounting evidence suggested that aberrantly expressed circRNAs are implicated in malignant tumors progression by competitive endogenous RNAs (ceRNAs) mechanism 8, 9. In HCC, the vital roles of circRNAs have been widely explored. Luo et al. found that circPTPRM expression was significantly high and could promote tumorigenesis and metastasis in HCC 10. Wang et al showed that circRHOT1 could promote the progression of HCC by increasing the expression of NR2F6 11. Tu et al. reported that the hsa_circ_0070269/miR-182/NPTX1 axis promoted HCC tumorigenesis 12. However, the roles of hsa_circ_0039053 in HCC remain uninvestigated.

MicroRNAs (miRNAs) are small ncRNAs that modulate gene expression via binding the 3'UTR of their specific mRNA 13, 14. MiR-637 has been verified to be associated with the malignant characteristics of tumors. Que et al. reported that decreased miR-637 expression was linked to poor prognosis and suppressed glioma cell progression through regulating Akt1 15. Li et al. found that miR-637 inhibited cholangiocarcinoma progression via targeting CTSB 16. Moreover, Zhang et al. found that miR-637 functioned by suppressing tumorigenesis in HCC by suppression the activation of Stat3 17. Nonetheless, the exact mechanisms and functions of miR-637 in HCC are unknown.

Here, we discovered a novel circRNA hsa_circ_0039053 in HCC. Next, we determined the biological roles, as well as the molecular pathways of hsa_circ_0039053 on HCC tumorigenesis. Our findings provided the hsa_circ_0039053/miR-637/USP21 regulatory network in HCC, which provided a novel therapeutic target.

## Materials and Methods

### Tissues collection

The expression profile of circRNAs in HCC was downloaded from the GEO database (GSE97332 and GSE94508). A total of 61 paired HCC tissues were obtained from patients at First Affiliated Hospital and College of Clinical Medicine of Henan University of Science and Technology. Only the patients who had not been subjected to radiotherapy or chemotherapy were included. The samples were preserved for subsequent analyses by snap freezing them in liquid nitrogen then storing at -80 °C. All subjects agreed to participate in the study and assigned informed consent (Table 1).

### Cell culture and transfection

Human cell lines of HCC (HepG2, SMMC-7721, Huh-7, Hep3B, and HCCLM3) and human hepatocyte cells (LO2) were procured from ATCC and grown in DMEM Medium (Gibco, Gaithersburg, MD, USA) containing 100 μg/mL streptomycin, 100 IU/mL 10% penicillin, and 10% FBS at 37 °C and 5% CO2.

siRNAs (small interfering RNAs) targeting hsa_circ_0039053 (si-circ_00390531#1: ATTTCATTTCCCGCTCCCGGC; si-circ_00390531#2: TCATTTCATTTCCCGCTCCCG; si-circ_00390531#3: CTCATTTCATTTCCCGCTCCC) and scrambled negative control (si-NC) were made by GenePharma (Shanghai, China). MiR-637 inhibitors and mimics, plus their negative controls, were designed by GeneCopoeia (Guangzhou, China). Lipofectamine 2000 reagents (Invitrogen, Grand Island, NY, USA) was utilized to transiently transfect oligonucleotides (50 nM) into cells.

### Quantitative real-time PCR

RNA isolation was executed utilizing TRIzol (Invitrogen) in reference to the protocols of manufacturers. After the RNAs were quantified, cDNA was generated with Primer Script™ (Takara, Dalian, China) RT reagent kit or TaqMan (Thermo Fisher Scientific, Waltham, MA, USA) microRNA Assay kit. Then qRT-PCR was manipulated with SYBR Premix Ex Taq II (Takara) and specific primers (GeneCopoeia). Thermal cycler parameters were 95 °C for 5 s, 45 cycles of 95 °C for 5 s, 60 °C for 10 s and 72 °C for 10 s, and extension at 72 °C for 5 min. Relative expression was calculated by the 2^-ΔΔCt^ method.

### Actinomycin D (Act D) assay and RNase R digestion assay

To prevent gene transcription, HCC cells were subjected to 2 μg/mL Act D (Sigma) or DMSO (Sigma) treatment at specified time points. Afterward, total RNA was isolated, followed by the determination of hsa_circ_0039053, and linear ITGAL levels.

For RNase R treatment, total RNA (5 µl) was digested with 10 U of RNase R (Epicentre, Madison, Wisconsin, USA) for 30 min at 37 °C. Next, the expression of hsa_circ_0039053 and linear ITGAL was estimated through qRT-PCR analysis.

### Subcellular fraction assay

Nuclear-cytoplasmic fractionation was conducted using Nuclear and Cytoplasmic Extraction Reagents PARIS™ Kit (Invitrogen) as per the methods described by the manufacturer and previous study 18.

### Cell proliferation assay

We employed the CCK-8 Kit (Dojindo, Japan) to determine the proliferative capacity of the cells. Transfected HCC cells (3000 cells/well) were seeded into 96-well plates. In total, 10μl CCK-8 reagent was added to the wells at various times following incubation. A microplate reader (Bio-Rad, USA) was utilized to determine the absorbance at 450 nm.

Moreover, cells were subjected to colony formation assays to further determine the proliferative capacity of the cells. Briefly, transfected HCC cells were seeded into a 6-well plate and cultured for 10 days. Then, colonies were rinsed thrice with PBS, fixed with methanol and stained with 0.1% crystal violet (Sigma).

### EdU and transwell invasion assays

Cells proliferation and invasion were analyzed using 5-ethynyl-2ʹ-deoxyuridineassay Kit (EdU, RiboBio) and transwell invasion assays according to previous study 11.

### Dual-luciferase reporter assay

The sequences of hsa_circ_0039053 (or 3'UTR of USP21), including the binding sequences of mutant or wild type miR-637, were cloned into pmirGLO plasmid (Promega), generating hsa_circ_0039053-WT, hsa_circ_0039053-MUT, USP21 3'UTR WT, and USP21 3'UTR MUT. The constructed luciferase reporter vector was transfected into cells alongside miR-637 mimics or miR-NC. Dual-Luciferase Reporter Assay Kit (Promega) was employed to quantify the luciferase activity.

### RNA Immunoprecipitation (RIP) assay

The cell lysates were gathered utilizing RIP lysis buffer. Following, they were subjected to conjugation with anti-Ago2 antibody in magnetic beads. Furthermore, anti-IgG antibody served as control. When immunoprecipitation was accomplished, qRT-PCR was adopted to quantify RNA enrichment.

### *In vivo* tumorigenesis in nude mice

Ten female BALB/c nude mice (4-week-old) were procured from Beijing HFK Bioscience Co., Ltd (China, Beijing). Transfect cells (1 × 106 cells) were intraperitoneally injected into mice (n = 5 per group). Tumor volumes (0.5 × length × width2) were detected weekly. At the 7th week post-injection, mice were sacrificed by cervical dislocation. Tumors were immediately removed and weighed.

### Data analysis

The SPSS 20.0 software was used to conduct the analyses. Data are displayed as mean ± SD. The means of 2 groups were compared using Student's t-test. Means of multiple groups were compared with one-way ANOVA. *P* ≤ 0.05 was regarded as statistically significant difference.

## Results

### Hsa_circ_0039053 was increased in HCC

**Figure 1:**
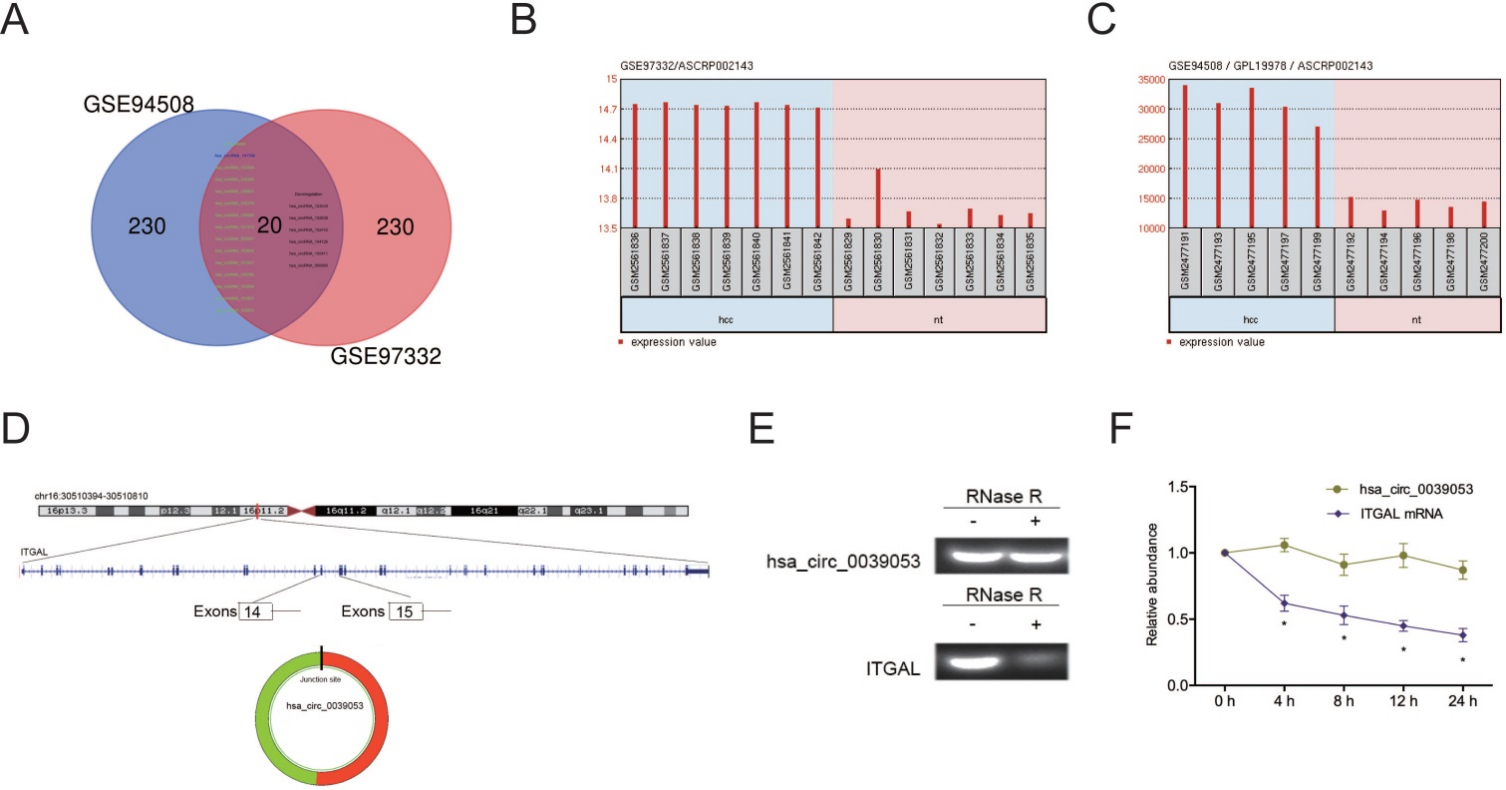
CircRNA expression profiles in HCC. (A) The Venn diagram overlapping sections show circRNAs that are dysregulated in the two microarray datasets (GSE94508 and GSE97332). (B, C) Hsa_circ_0039053 expression in GSE94508 and GSE97332. (D) Schematic illustration of hsa_circ_0039053. (E) QRT-PCR was utilized to evaluate the resistance of linear ITGAL and hsa_circ_0039053 to RNase R in cells. (F) The stability of hsa_circ_0039053 and ITGAL mRNA in cells was explored by Actinomycin D assay. *P < 0.05.

To elucidate the functions of circRNAs in HCC progression, we analyzed two studies on circRNA expression in HCC (GSE94508 and GSE97332). Twenty circRNAs (14 upregulation, 6 downregulation) were found to be aberrantly expressed in both studies (Figure 1A-C). We then focused on one of the most upregulated circRNA hsa_circ_0039053 (circITGAL) located on chromosome 16, and consists of 2 exons (exons 14-15) from its host gene ITGAL (Figure 1D). Then, we used RNase R to digest total RNAs and found that hsa_circ_0039053 was significantly resistant to RNase R relative to linear ITGAL, suggesting hsa_circ_0039053 is circular (Figure 1E). Actinomycin D assay was further confirmed the results (Figure 1F).

**Figure 2:**
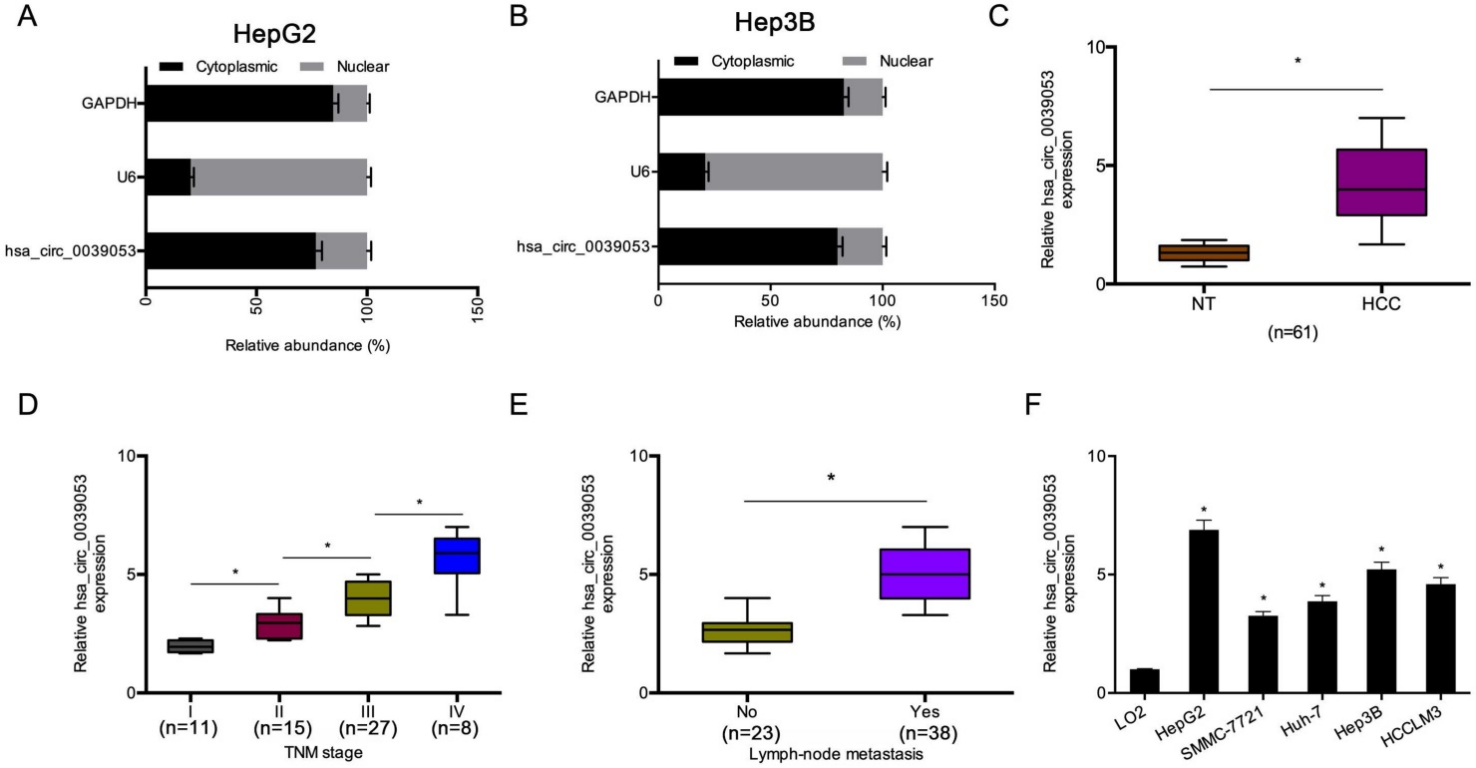
Hsa_circ_0039053 was increased in HCC. (A, B) Levels of hsa_circ_0039053 in the cytoplasmic and nuclear fractions of HCC cells were explored by a subcellular fraction assay. (C) Hsa_circ_0039053 was dramatically upregulated in HCC tissues. (D, E) High hsa_circ_0039053 expression was linked to advanced TNM stage and lymph node metastasis. (F) Hsa_circ_0039053 was increased in HCC cell lines. *P < 0.05.

Then, we explored the cellular localization of hsa_circ_0039053. The cell subcellular fraction assay indicated that hsa_circ_0039053 was preferentially localized to the cytoplasm and was hardly expressed in the nucleus (Figure 2A-B). Afterward, we examined the expression of hsa_circ_0039053 in HCC. Gene expression analysis (qRT-PCR) indicated that hsa_circ_0039053 expression was remarkably overexpressed and was linked to advanced TNM stage, as well as lymph node metastasis in HCC patients (Figure 2C-E). In addition, hsa_circ_0039053 was also upregulated in HCC cells (HepG2, SMMC-7721, Huh-7, Hep3B, HCCLM3) (Figure 2F). Thus, these data suggested that hsa_circ_0039053 was overexpressed in HCC.

### Hsa_circ_0039053 promote HCC cells progression

**Figure 3:**
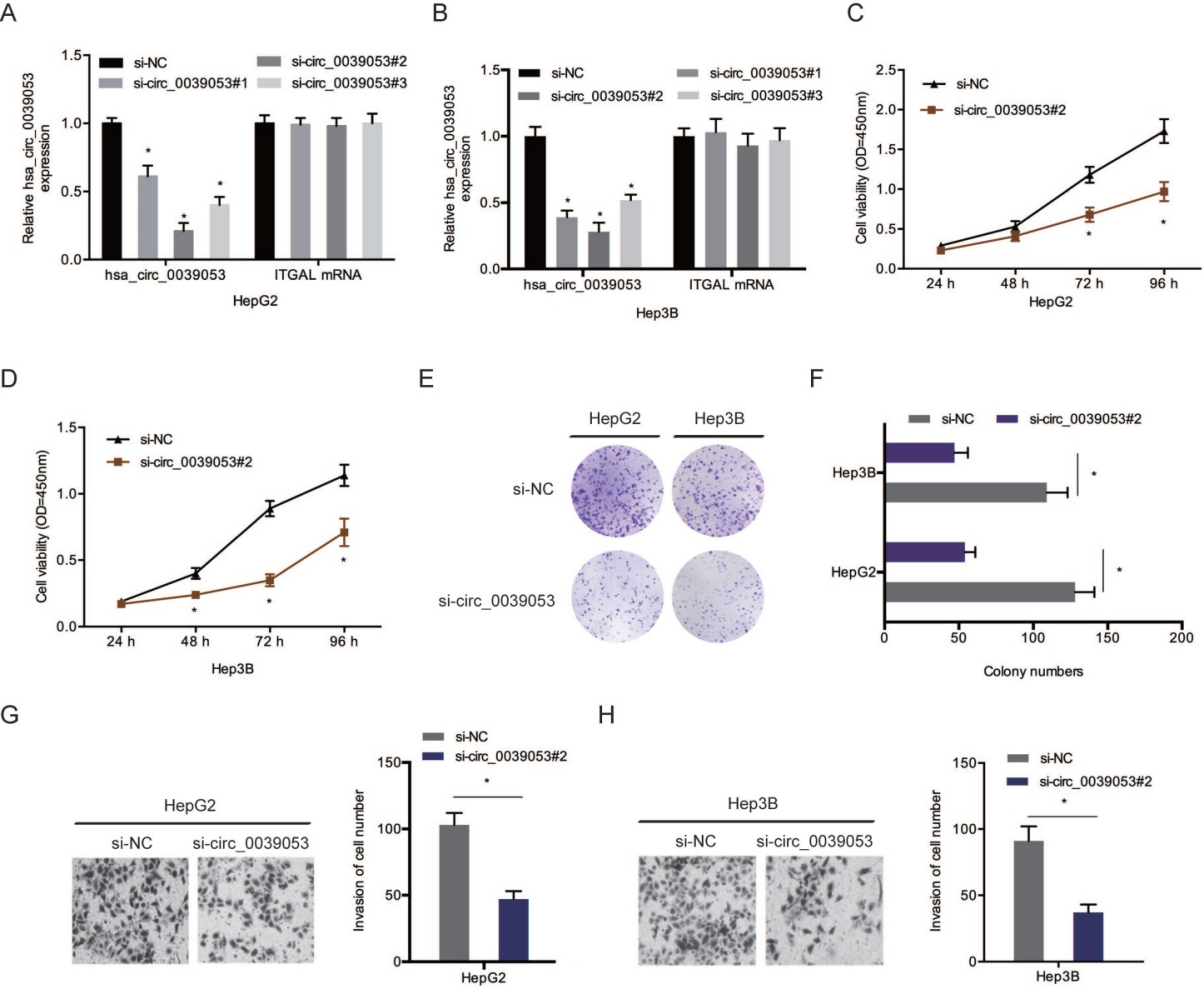
Hsa_circ_0039053 enhanced the invasion and proliferative abilities of HCC cells. (A, B) Interference efficiency of hsa_circ_0039053 knockdown in HCC cells. (C-F) The proliferation abilities of HCC cells were assessed by CCK-8 and colony formation assays. (G, H) Transwell assay was used to evaluate HCC cells invasion capacity. *P < 0.05.

To verify the roles of hsa_circ_0039053 in HCC progression, si-circ_0039053 was transfected into HCC cells (Figure 3A and 3B). CCK-8 and colony formation analyses showed that silencing of hsa_circ_0039053 led to a pronounced inhibition of the proliferation of HCC cells *in vitro* (Figure 3C-F). Transwell assay indicated that hsa_circ_0039053 interference conspicuously reduced the invasion capacity of HCC cells *in vitro* (Figure 3G and 3H).

**Figure 4:**
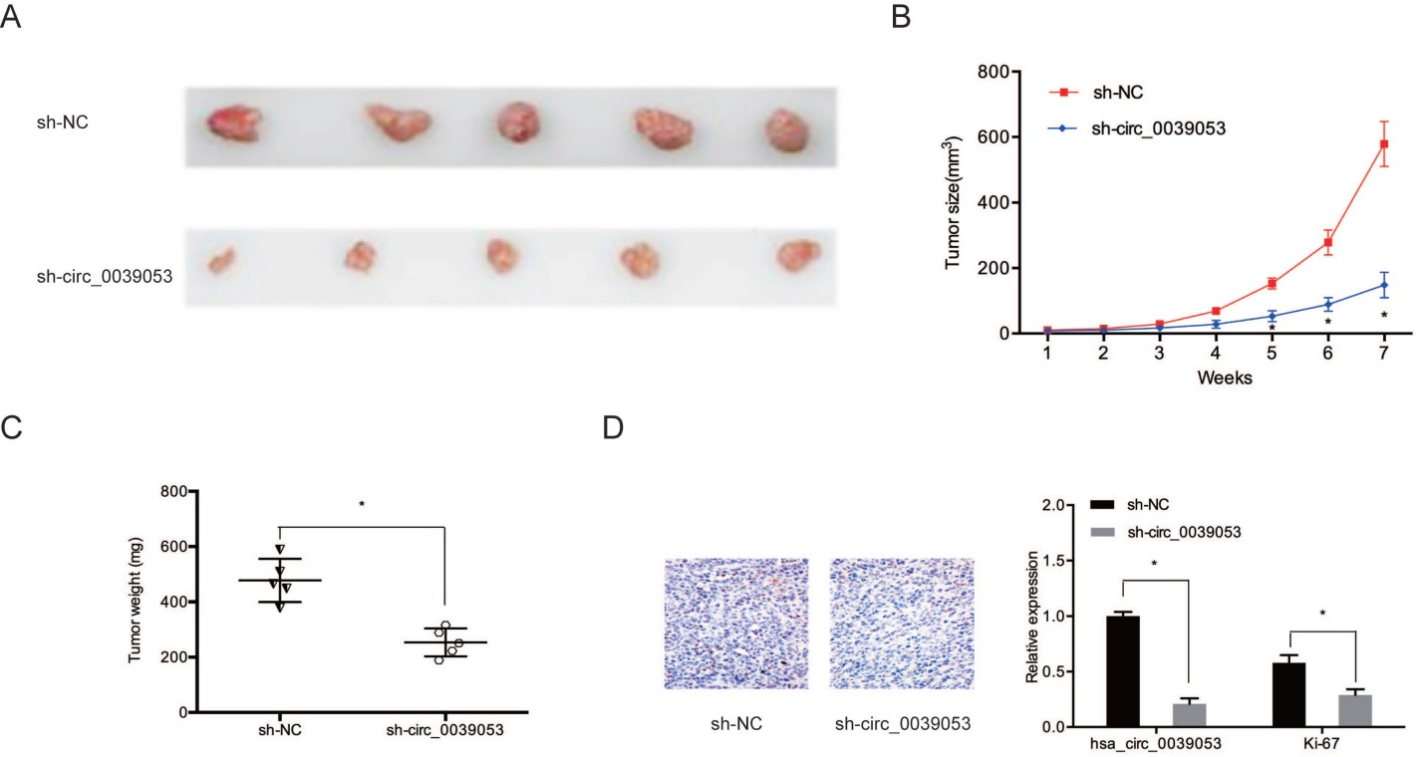
Hsa_circ_0039053 increased HCC growth *in vivo*. (A) Image of subcutaneous tumor tissues. (B, C) The tumor volume and weights of subcutaneous xenograft tumors from nude mice. (D) Ki-67 expression was observed in subcutaneous tumor tissues by IHC. *P < 0.05.

Then, we determined the impact of hsa_circ_0039053 on tumor progression *in vivo*. Results showed that hsa_circ_0039053 silencing notably suppressed tumor progression *in vivo* (Figure 4A and 4B). Meanwhile, the weights of tumors in the hsa_circ_0039053 suppression group were lower relative to control group (sh-NC) (Figure 4C). Immunohistochemistry (IHC) showed that Ki-67 expression levels were reduced in the hsa_circ_0039053 knockdown group in comparison to the sh-NC group (Figure 4D). These findings indicated that hsa_circ_0039053 knockdown delayed the proliferation of HCC cells, both *in vivo* and* in vitro.*

### Hsa_circ_0039053 sponged miR-637 in HCC

**Figure 5:**
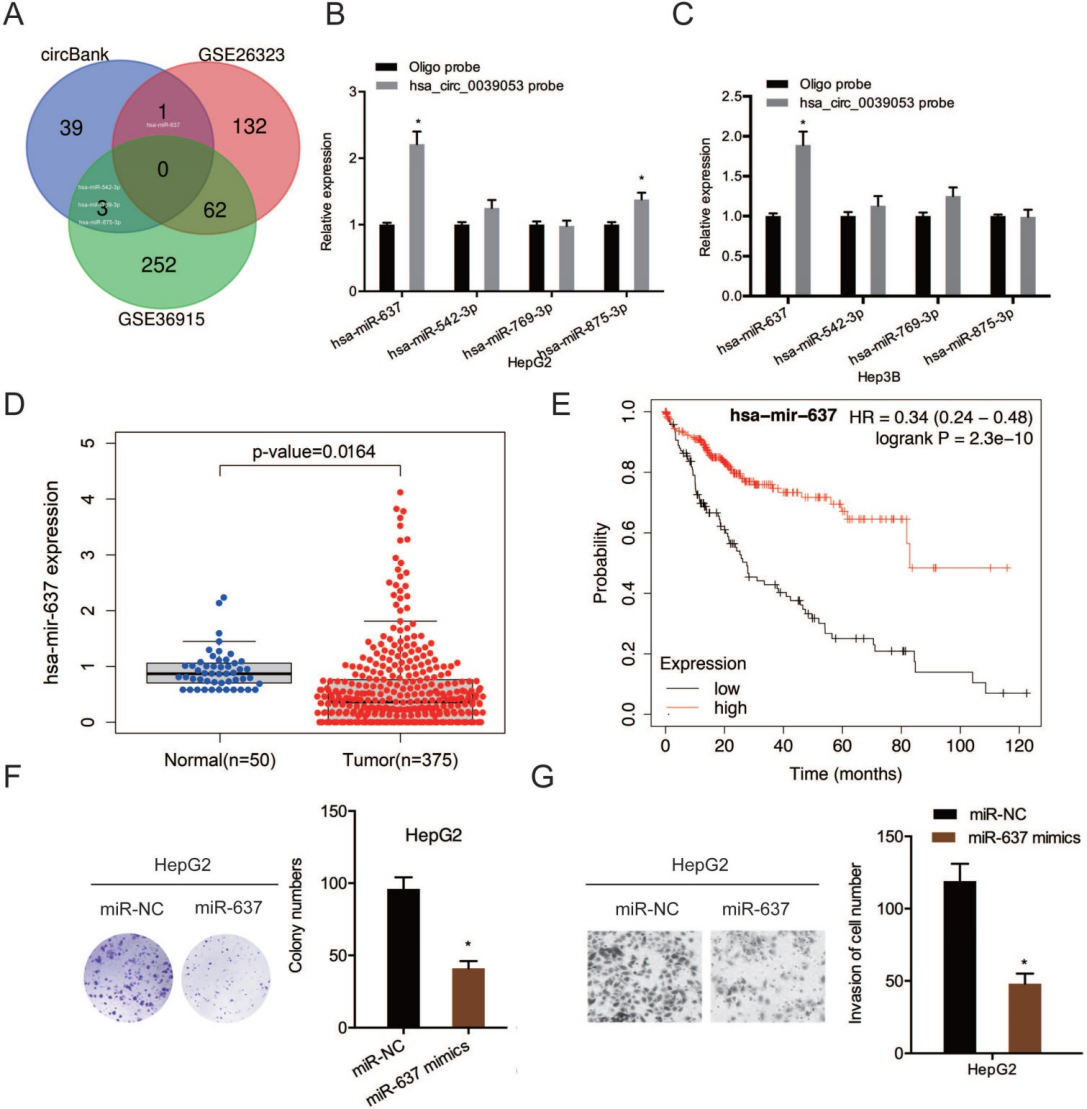
MiR-637 targeted hsa_circ_0039053. (A) Schematic illustration exhibiting the overlapping of the target miRNAs of hsa_circ_0039053 predicted by circBank, and dbDEMC 2.0 (GSE26323 and GSE36914). (B, C) QRT-PCR showing the expression of potential miRNAs target in HCC cells. (D) MiR-637 expression in the TCGA database. (E) Low miR-637 expression was linked to poor overall survival in HCC patients. (F, G) MiR-637 mimics reduced HCC cells proliferation and invasion abilities *in vitro*. *P < 0.05.

To examine further the molecular mechanism of hsa_circ_0039053 in regulating the malignant behaviors of HCC, we screened the potential target miRNAs by searching bioinformatics analysis software circBank and dbDEMC 2.0 (GSE26323 and GSE36914), we found that only four miRNAs (hsa-miR-637, hsa-miR-542-3p, hsa-miR-769-3p, hsa-miR-875-3p) existed in circBank and dbDEMC 2.0 (Figure 5A). RNA pull-down assay revealed that hsa_circ_0039053 probe could pull down more miR-637 than other miRNAs in HCC cells (Figure 5B and 5C). Next, based on TCGA database, miR-637 expression was remarkably reduced in HCC, and this was linked to poor overall survival in patients (Figure 5D and 5E). Function assays showed that miR-637 mimics reduced HCC cells proliferation and invasion abilities *in vitro* (Figure 5F and 5G). Thus, we selected miR-637 as our research object.

**Figure 6:**
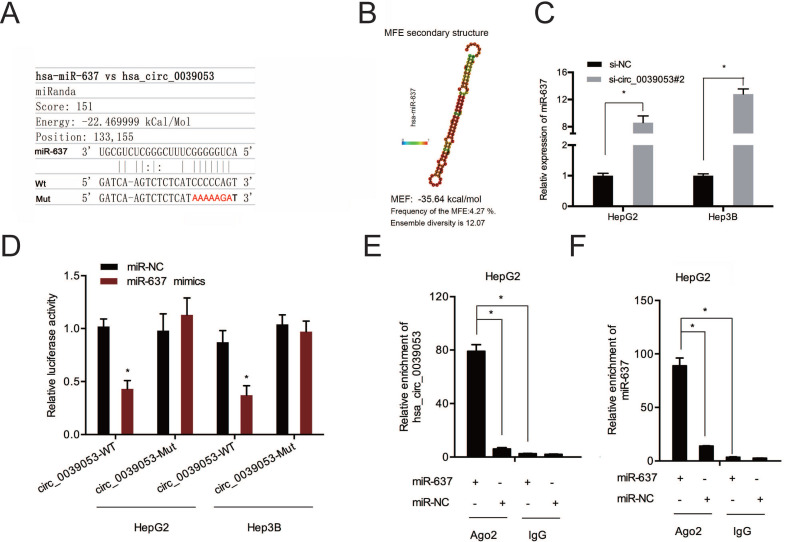
Hsa_circ_0039053 sponged miR-637 in HCC. (A, B) Schematic representation of the miR-637 site in hsa_circ_0039053. (C) Hsa_circ_0039053 knockdown increased miR-637 expression in HCC cells. (D) Luciferase activity in HCC cells co-transfected with miR-637 mimics and luciferase reporters containing mutant or wild-type hsa_circ_0039053. (E, F) RIP assay revealed that hsa_circ_0039053 and miR-637 were enriched in the Ago2 platform. *P < 0.05.

Next, the complementary sequences of hsa_circ_0039053 and miR-637 were shown in Figure 6A and 6B. QRT-PCR showed that hsa_circ_0039053 knockdown notably enhanced miR-637 expression in HCC cells (Figure 6C). Luciferase reporter analysis found that miR-637 upregulation led to a remarkable suppression of luciferase activity in the wild-type (Wt) group (Figure 6D). And RIP assay further confirmed the association between hsa_circ_0039053 and miR-637 in HepG2 cells (Figure 6E and 6F). Collectively, hsa_circ_0039053 negatively regulated miR-637 expression by directly targeting.

### USP21 is a downstream miR-637 target in HCC

**Figure 7:**
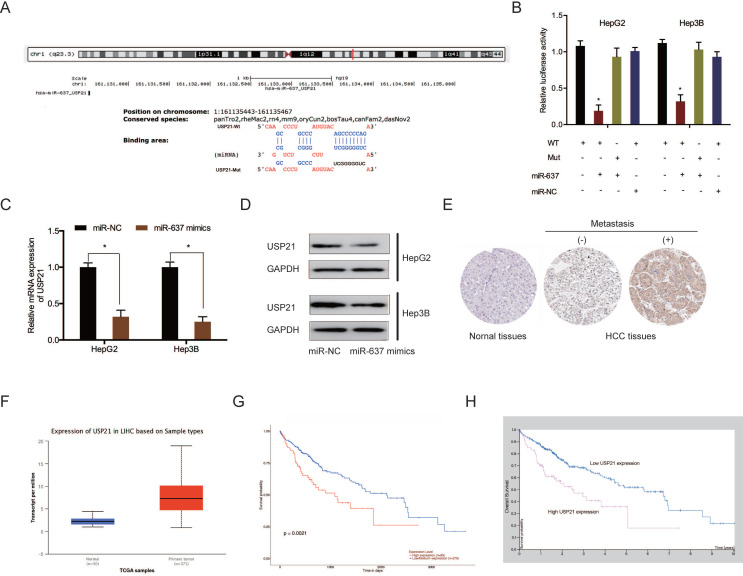
MiR-637 directly targeted USP21. (A) Schematic representation of the miR-637 site in USP21. (B) Luciferase reporter assay was operated to confirm the interaction between miR-637 and USP21. (C, D) MiR-637 mimics reduced USP21 levels in HCC cells. (E) The protein levels of USP21 in HCC tissues were measured by IHC. (F) USP21 expression in HCC explored by TCGA database. (G, H) High USP21 expression was linked to poor disease outcomes in HCC patients. *P < 0.05.

Subsequently, we investigated the potential target genes of miR-637. Bioinformatics analysis showed that USP21 3'UTR contained the putative binding sequences of miR-637 (Figure 7A). Dual-luciferase reporter assay displayed that miR-637 mimics decreased the luciferase activity of USP21 3'UTR WT, whereas no change was found in other groups (Figure 7B). QRT-PCR and western blot assays indicated that miR-637 mimics apparently reduced USP21 levels in HCC cells (Figure 7C and 7D). Moreover, we explored USP21 levels in HCC. IHC showed that USP21 protein levels were upregulated in HCC tissues (Figure 7E). These results were verified further by the TCGA database (Figure 7F). Kaplan-Meier analysis indicated that USP21 upregulation was significantly related to poor disease outcomes in HCC patients (Figure 7G and 7H). These findings illustrated that miR-637 inversely modulated USP21 expression in HCC.

### Hsa_circ_0039053 promoted HCC proliferation and invasion via the miR-637/ USP21 axis

**Figure 8:**
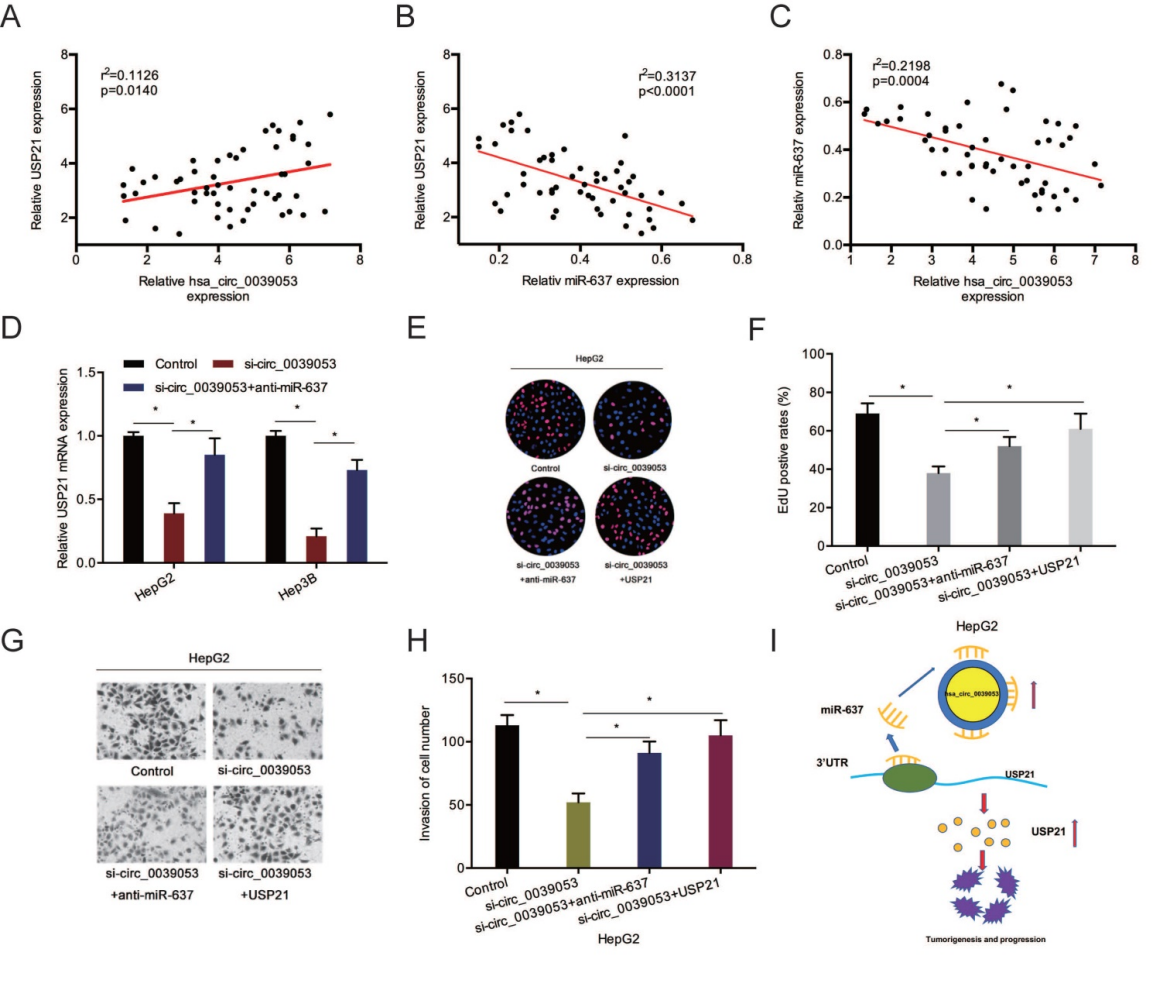
Hsa_circ_0039053 elevated USP21 expression via sponging miR-637. (A-C) Spearman's correlation coefficient was carried out to analyze the linear correlation among hsa_circ_0039053, miR-637, and USP21 in HCC tissues. (D) MiR-637 inhibitors abolished the impacts of hsa_circ_0039053 inhibition on USP21 expression in HCC cells. (E-H) SP21 overexpression (miR-637 inhibition) abolished the roles of hsa_circ_0039053 silencing on HCC cells proliferation and invasion abilities. (I) A schematic diagram of the hsa_circ_0039053/miR-637/USP21 axis in HCC progression. *P < 0.05.

To further confirm the hsa_circ_0039053/miR-637/USP21 axis in HCC, we firstly determined the relationships between them. Association assays revealed that USP21 expression was positively linked to hsa_circ_0039053 expression and inversely related to miR-637 expression (Figure 8A and 8B). And hsa_circ_0039053 expression was inversely related to miR-637 expression (Figure 8C). Next, gene expression (qRT-PCR) assay revealed that hsa_circ_0007364 suppression markedly reduced USP21 mRNA expression, while miR-637 inhibitors abolished this effect (Figure 8D). Rescue assays showed that USP21 overexpression or miR-637 inhibition reversed the roles of hsa_circ_0039053 silencing on HCC cell proliferation and invasion capacities *in vitro* (Figure 8E-H). These outcomes indicated that hsa_circ_0039053 might enhance HCC progression by miR-637 sponging to regulate USP21 expression (Figure 8I).

## Discussion

Increasing evidence has uncovered that aberrant expression of circRNAs is associated with malignant phenotypes 2, 3. With the knowledge of the roles of circRNAs in HCC, a multitude of novel biomarkers related to carcinogenesis have been exposed in recent years 10-12. In the present study, we analysis two GEO dataset (GSE94508 and GSE97332) and reported a novel circRNA hsa_circ_0039053, which was obviously upregulated in HCC cell lines and tissues. Hsa_circ_0039053 (circITGAL) was derived from the integrin subunit alpha L (ITGAL), which has been reported play a central role in leukocyte intercellular adhesion 19, 20. Herein, we are the first to demonstrate that hsa_circ_0039053 deregulation inhibits the invasion and proliferative abilities of HCC cells and suppresses the progression of the tumor *in vivo*. These findings show that hsa_circ_0039053 promotes HCC development.

Circular RNAs sponge miRNAs to regulate their functions 21, 22. Herein, we revealed that hsa_circ_0039053 could serve as the ceRNA of miR-637 to increase USP21 expression in HCC. Previous studies demonstrated that miR-637 exerted the tumor-inhibitory roles in diverse human tumors, such as cervical cancer, colorectal cancer, and gastric cancer 23-25. In consistence of previous studies, we also observed that miR-637 was reduced in HCC and the elevation of miR-637 was linked to poor disease outcomes. MiR-637 overexpression reduced HCC cells progression. Moreover, miR-637 suppression abolished the impacts of hsa_circ_0039053 interference on the metastatic features of HCC cells *in vitro*. These findings illustrated that hsa_circ_0039053 aggravated HCC progression by sponging miR-637.

USP21 belongs to the ubiquitin-specific peptidases (USPs) subfamily and plays important functions in tumorigenesis 26. Chen et al. reported that USP21 enhanced the metastatic and proliferative ability of bladder cancer by regulating EZH2 27. Xu et al. showed that the USP21/YY1/SNHG16 axis enhances the progression of NSCL 28. A study by Liu et al. found that lncRNA FGD5-AS1 regulates oral squamous cell carcinoma progression by interacting with the miR-520b/USP21 axis 29. In line with these reports, we found that USP21 was raised in HCC and promoted HCC progression by targeting miR-637. Moreover, we showed that USP21 overexpression abrogated the suppressive impacts of hsa_circ_0039053 inhibition on the malignant behaviors of HCC.

In summary, our work identified hsa_circ_0039053 as a cancerogenic molecule in regulating HCC cell proliferation, and invasion on the basis of the regulation of the miR-637/USP21 regulatory axis, providing a biomarker and a possible target for HCC treatment.

## Figures and Tables

**Table 1:** The characteristics of HCC patients

Characteristics		Number
Gender	Male	39
	Female	22
Age	<50	21
	≥50	40
Tumor size (cm)	<5	34
	≥5	27
TNM stage	I/II	26
	III/IV	35
Lymph-node metastasis	No	23
	Yes	38
Cirrhosis history	Positive	48
	Negative	13
